# Allostimulatory capacity of conditionally immortalized proximal tubule cell lines for bioartificial kidney application

**DOI:** 10.1038/s41598-017-07582-1

**Published:** 2017-08-02

**Authors:** Milos Mihajlovic, Lambertus P. van den Heuvel, Joost G. Hoenderop, Jitske Jansen, Martijn J. Wilmer, Annemarie J. F. Westheim, Wil A. Allebes, Dimitrios Stamatialis, Luuk B. Hilbrands, Rosalinde Masereeuw

**Affiliations:** 1 Division of Pharmacology, Utrecht Institute for Pharmaceutical Sciences, Utrecht University, Utrecht, The Netherlands; 2 Department of Pediatric Nephrology, Radboud university medical center, Nijmegen, The Netherlands; 3 Department of Physiology, Radboud Institute for Molecular Life Sciences, Radboud university medical center, Nijmegen, The Netherlands; 4 Department of Pharmacology and Toxicology, Radboud Institute for Molecular Life Sciences, Radboud university medical center, Nijmegen, The Netherlands; 5 Department of Laboratory Medicine, Laboratory for Medical Immunology (LMI), Radboud university medical center, Nijmegen, The Netherlands; 6 Department of Biomaterials Science and Technology, MIRA Institute for Biomedical Technology and Technical Medicine, University of Twente, Enschede, The Netherlands; 7 Department of Nephrology, Radboud university medical center, Nijmegen, The Netherlands

## Abstract

Novel renal replacement therapies, such as a bioartificial kidney (BAK), are needed to improve current hemodialysis treatment of end-stage renal disease (ESRD) patients. As BAK applications may reveal safety concerns, we assessed the alloimmunization and related safety aspects of readily available conditionally immortalized human proximal tubule epithelial cell (ciPTEC) lines to be used in BAK. Two ciPTEC lines, originally derived from urine and kidney tissue, were characterized for the expression and secretion of relevant molecules involved in alloimmunization and inflammatory responses, such as HLA class-I, HLA-DR, CD40, CD80, CD86, as wells as soluble HLA class I and proinflammatory cytokines (IL-6, IL-8 and TNF-α). A lack of direct immunogenic effect of ciPTEC was shown in co-culture experiments with peripheral blood mononuclear cells (PBMC), after appropriate stimulation of ciPTEC. Tight epithelial cell monolayer formation on polyethersulfone flat membranes was confirmed by zonula occludens-1 (ZO-1) expression in the ciPTEC tight junctions, and by restricted inulin-FITC diffusion. Co-culture with (activated) PBMC did not jeopardize the transepithelial barrier function of ciPTEC. In conclusion, the absence of allostimulatory effects and the stability of ciPTEC monolayers show that these unique cells could represent a safe option for BAK engineering application.

## Introduction

End-stage renal disease (ESRD) is the final and most severe stage of chronic kidney disease (CKD). It has been estimated that almost 10% of the population worldwide is affected by CKD. The major problem in CKD patients, beside the loss of kidney function, is the concomitant presence of various comorbidities, especially cardiovascular disorders. These develop over time as a result of longstanding hypertension, disturbances in calcium-phosphate metabolism, and constant accumulation of uremic metabolites, and result in increased mortality within the CKD population^1–3^. Currently available treatment options for patients with ESRD are hemodialysis, peritoneal dialysis and kidney transplantation, the latter one being preferred since the ability to restore kidney function is associated with a better life expectancy and a higher quality of life. Unfortunately, for many ESRD patients this treatment is not readily available because of organ shortage, which keeps these patients dependent on dialysis. However, dialysis is not very efficient in removing the uremic waste products, especially the protein-bound and larger size molecules, maintaining the progression of most of the mortality-associated comorbidities^4^.

Therefore, novel therapies for CKD are needed, and one of the most promising options is the bioartificial kidney (BAK) device, composed of proximal tubule epithelial cells (PTEC) cultured on hollow fiber membranes (HFM) with formation of confluent, fully differentiated epithelial monolayers^5^. The reason why PTEC are especially attractive for such an application is that these cells are specialized in the excretion of many xenobiotics, including the endogenous uremic waste compounds (also named uremic toxins). In particular, PTEC can help to excrete protein-bound toxins, which cannot be removed by standard dialysis treatments. Recent work from our group showed that PTEC cultured on HFM are able to take up and excrete indoxyl sulfate and kynurenic acid, two prototypical protein-bound uremic toxins^6^.

One of the crucial issues to take into consideration when developing a BAK is sufficient availability of suitable cells. We developed conditionally immortalized proximal tubule epithelial cell lines (ciPTEC), derived from human urine or kidney tissue, as an unlimited and invariable cell source for BAK application^7, 8^. ciPTEC were immortalized with the temperature-sensitive mutant U19tsA58 of SV40 large T antigen (SV40T) and the essential catalytic subunit of human telomerase (hTERT), as described^9–11^. This allows the cells to proliferate at the permissive temperature of 33 °C and to fully differentiate to mature PTEC at non-permissive temperature of 37 °C. ciPTEC were extensively characterized for most proximal tubule functions such as reabsorption and excretory transport activities and successfully cultured on biofunctionalized HFM^6–8, 12^.

Many of the previous studies concerning BAK have focused on the immunomodulatory function of renal tubular cells, in particular reduction of pro-inflammatory and increase of anti-inflammatory cytokines plasma and serum levels^13–15^. In the present study, though, we evaluated the *in vitro* immunosafety of ciPTEC for BAK application, with particular attention to their direct allogeneic effect. To that purpose, we thoroughly characterized the expression and release of Human Leukocyte Antigens (HLA), as well as the expression of several co-stimulatory ligands on two ciPTEC lines, one originally derived from healthy donor urine and one from kidney tissue^7, 8^. In addition, we assessed the ability of ciPTEC to mediate an inflammatory response by measuring the production of relevant proinflammatory mediators, like Interleukin 6 (IL-6), Tumor Necrosis Factor α (TNF-α), and Interleukin 8 (IL-8), in various stimulatory conditions. In order to determine the direct immunogenic effect of ciPTEC, co-culture experiments with immune cells were performed. Finally, the paracrine effect of immune cells on ciPTEC monolayer integrity was examined as well. For this, cells were grown on flat biofunctionalized polyethersulfone membranes, with a Mw cut-off of 50 kDa, as prototypical component of a BAK device^6, 16–19^.

## Results

### HLA class I expression and release by ciPTEC-U and –T1 lines

**Figure 1:**
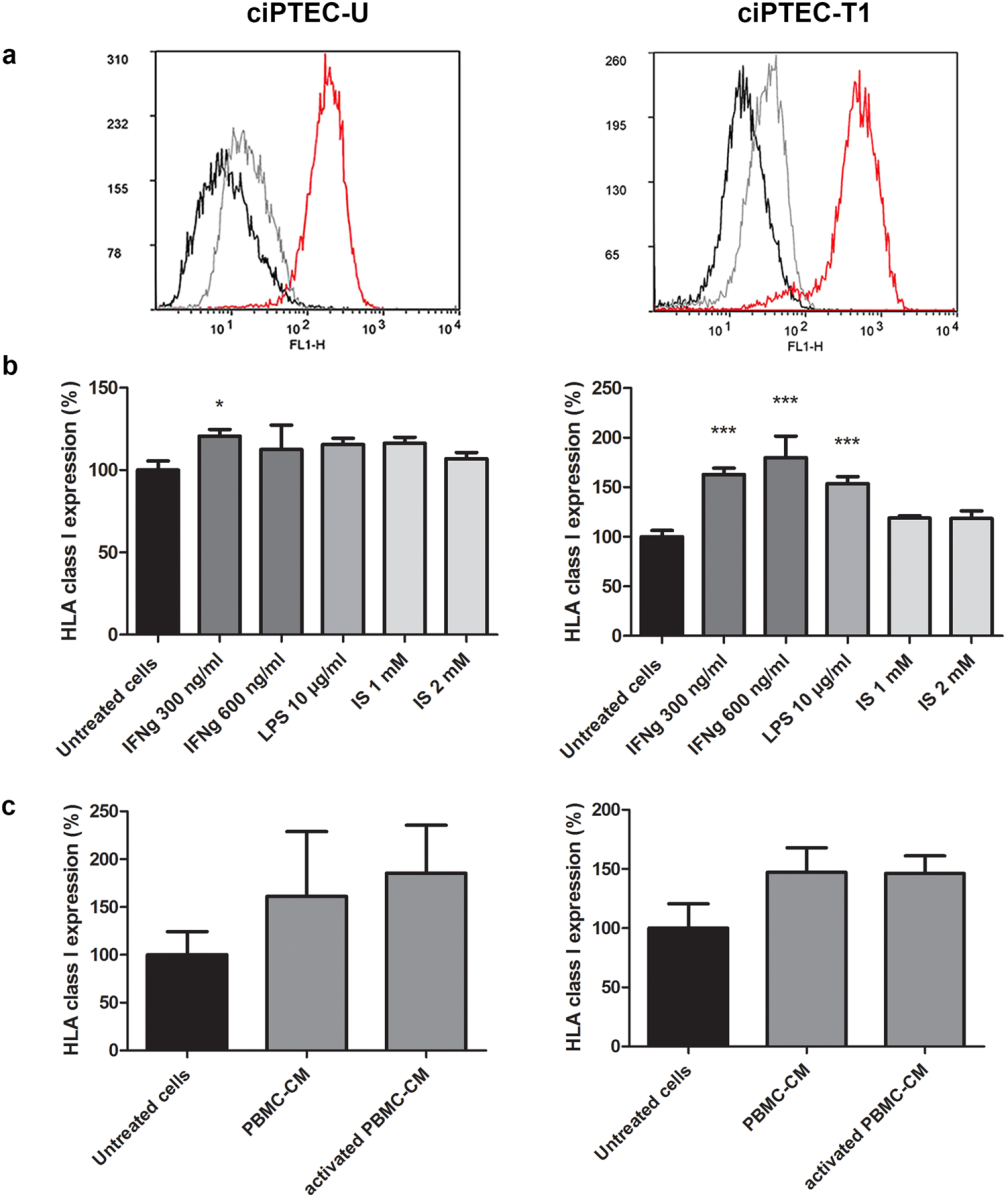
HLA class I expression in ciPTEC in various stimulatory conditions. (**a**) Representative histograms of HLA class I expression from flow cytometric analysis. Black histograms correspond to the unstained cells, grey to the negative control (absence of primary monoclonal W6/32 antibody), and red represent basal levels of HLA class I expression. (**b**) Effect of IFN-γ (300 and 600 ng/ml), LPS (10 µg/ml) and indoxyl sulfate (IS) (1 and 2 mM) on HLA class I expression in ciPTEC-U and -T1. Treatments were performed for 48 h prior to flow cytometric measurements. (**c**) Effect of conditioned medium derived from resting (PBMC-CM) and aCD3/aCD28 activated (activated PBMC-CM) PBMC on the expression of HLA class I after 48 h exposure. Expression is shown as %, based on median fluorescence intensity (MFI) and SEM values of three independent experiments performed in duplicate. *p < 0.05, ***p < 0.001, compared to untreated cells (One-way ANOVA, Dunnett’s multiple comparison test).

The HLA type of the two ciPTEC lines was determined by PCR-SSO. The results are summarized in Supplementary Table S1. Next, the surface expression of HLA class I molecules was evaluated in both ciPTEC-U and –T1 lines in basal, as well as in several stimulatory conditions, using W6/32 monoclonal antibody directed against the core of HLA-A, B and C antigens. Both cell lines showed the expression of HLA class I molecules in untreated conditions. In ciPTEC-U, the HLA class I expression was increased after stimulation with IFN-γ at 300 ng/ml for 48 h (21 ± 4%, p < 0.05). In ciPTEC-T1 the HLA class I expression was enhanced by IFN-γ at 300 and 600 ng/ml (63 ± 7% and 80 ± 22%, respectively, p < 0.001) and by LPS 10 µg/ml (54 ± 7%, p < 0.001). The expression of HLA class I molecules was not significantly upregulated after 48 h stimulation with indoxyl sulfate (1 and 2 mM), or conditioned medium (CM) from PBMC (resting and CD3/CD28 activated) (Fig. 1).

**Figure 2:**
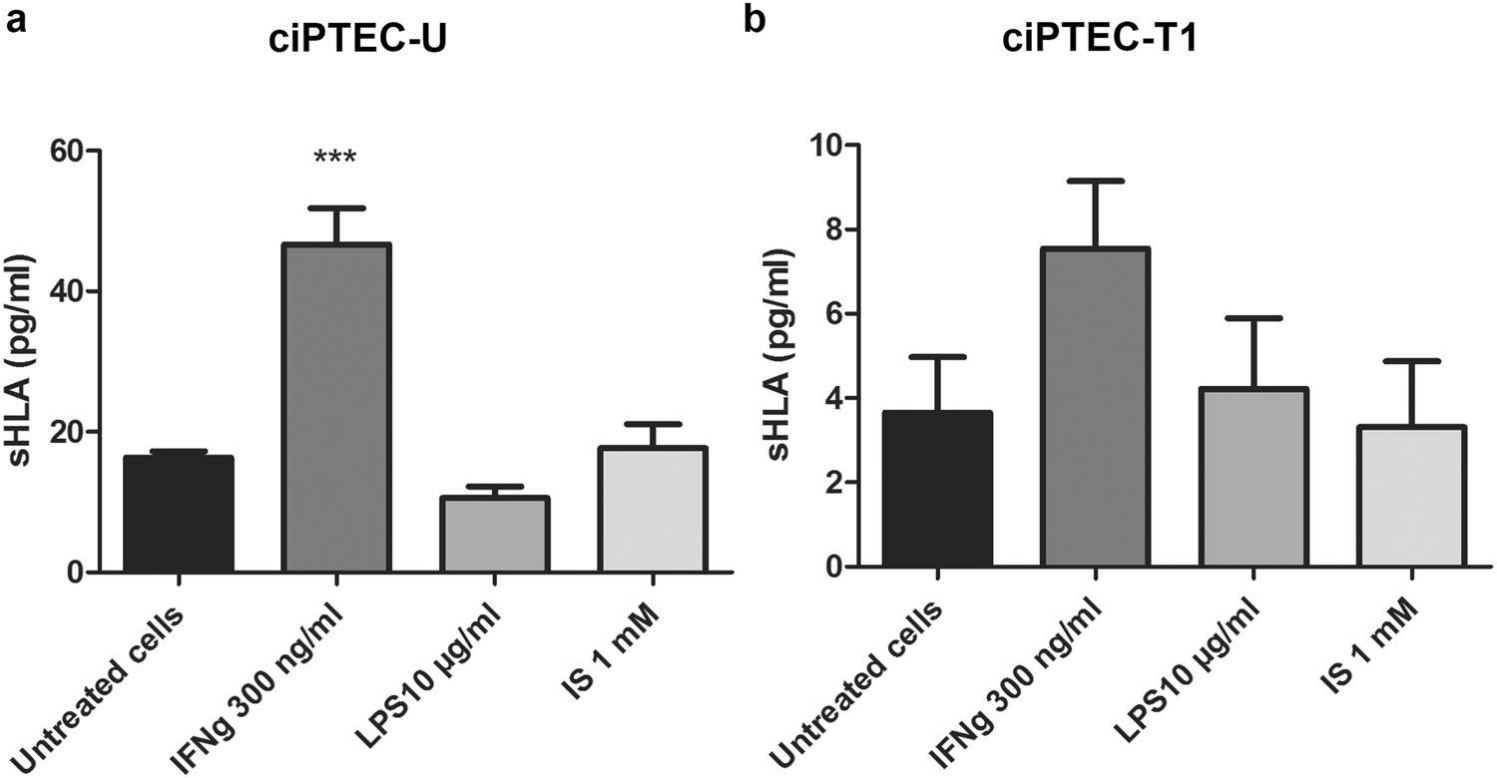
Release of soluble HLA (sHLA) class I antigens by ciPTEC. Secretion of sHLA class I molecules in cell culture supernatant following 48 h stimulation with IFN-γ (300 ng/ml), LPS (10 µg/ml) and indoxyl sulfate (IS) (1 mM) by (**a**) ciPTEC-U and (**b**) ciPTEC-T1, measured using ELISA assay and expressed as pg/ml (mean ± SEM). Two independent experiments were performed in duplicate. ***p < 0.001 compared to untreated cells (One-way ANOVA, Dunnett’s multiple comparison test).

In addition to surface expression, the release of soluble forms of HLA class I molecules in cell culture supernatants was also examined. We found low concentrations of sHLA class I in the supernatant of untreated cells, with a significant increase after stimulation with IFN-γ (300 ng/ml) in ciPTEC-U, and a similar trend in ciPTEC-T1 (Fig. 2). No increase in sHLA class I concentration was observed following LPS or indoxyl sulfate treatment.

### The expression of HLA class II and co-stimulatory molecules

**Figure 3:**
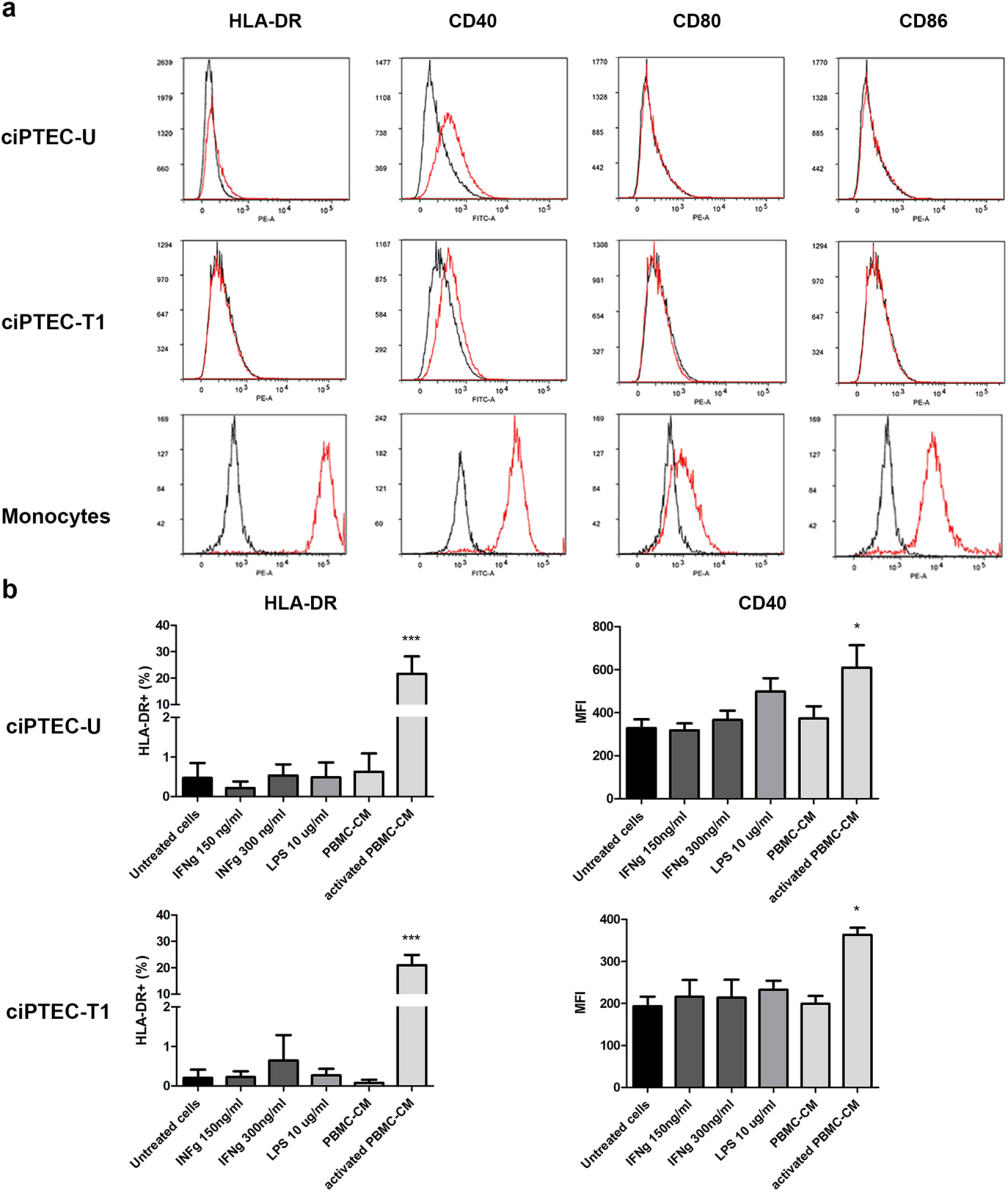
Expression of co-stimulatory molecules in ciPTEC. (**a**) Representative histograms from flow cytometric analysis of HLA-DR, CD40, CD80 and CD86 in ciPTEC-U, -T1 and monocytes (used here as a cell type positive for all four surface markers evaluated). In all histogram plots black color represents blank (unstained cells) and red color represents basal expression of either HLA-DR, CD40, CD80 or CD86. (**b**) Quantification of HLA-DR and CD40 expression in ciPTEC cell lines, expressed as % of positive cells ± SEM and MFI ± SEM respectively. Three independent experiments were performed. *p < 0.05, ***p < 0.001, compared to untreated control (One-way ANOVA, Dunnett’s multiple comparison test).

HLA-DR expression was measured as an indicator of the presence of HLA class II molecules on ciPTEC. In both ciPTEC-U and –T1, the expression of HLA-DR was undetectable in control conditions. Treatment with conditioned medium from activated PBMC (activated PBMC-CM) was the only tested stimulatory condition able to induce the expression of HLA-DR. Following 48 h exposure to activated PBMC-CM, 22 ± 7% of ciPTEC-U and 21 ± 4% of ciPTEC-T1 stained positive with anti HLA-DR antibody (Fig. 3). Other molecules that are important for the immunogenicity of allogeneic tissues and cells are the co-stimulatory ligands CD40, CD80 and CD86. We found a low expression of CD40, as compared to monocytes which were used as positive controls, on both cell lines. Again, the only condition tested able to induce an increase in CD40 expression was the activated PBMC-CM (Fig. 3). The expression of CD80 and CD86 was either very low or undetectable in all conditions examined.

### Lack of allostimulation by ciPTEC in direct co-culture with PBMC

**Figure 4:**
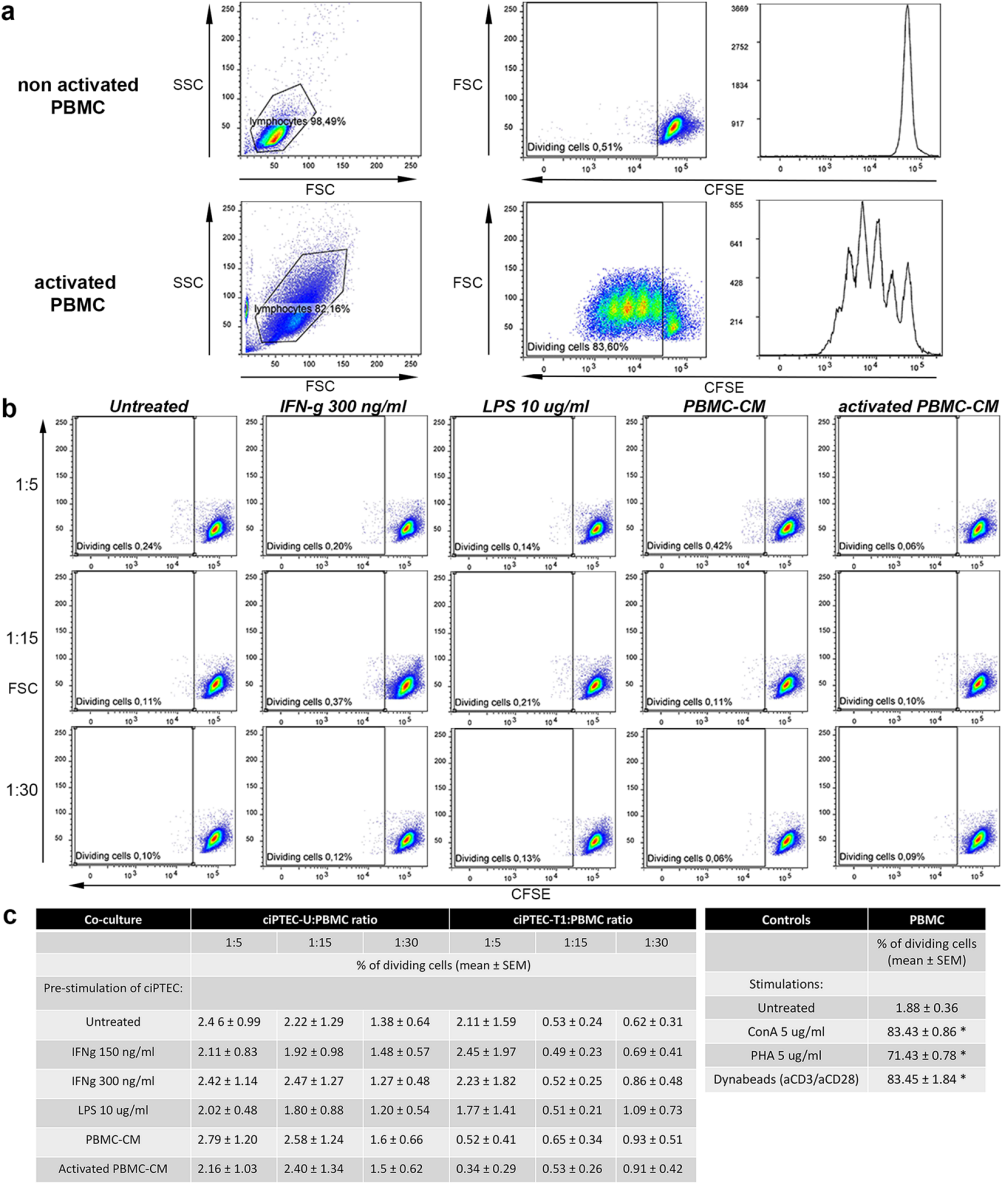
Immunogenic effect of ciPTEC in direct co-culture with PBMC. (**a**) Representative dot plots and histograms of lymphocyte proliferation assays that were performed using carboxyfluorescein diacetate succinimidyl ester (CFSE)-labeled PBMC either untreated or activated with human T-Activator CD3/CD28 dynabeads (1 bead per cell). (**b**) Representative dot plots of CFSE-labeled PBMC proliferation after 5 days of co-culture with ciPTEC-U (untreated or pre-stimulated in various ways) in ratios 1:5, 1:15 or 1:30. Comparable dot plots were observed for the co-culture of PBMC and ciPTEC-T1. (**c**) PBMC proliferation after 5 days of co-culture in various ratios with untreated or pre-stimulated ciPTEC-U and –T1 cells, expressed as % of dividing cells. There were no differences in proliferation of PBMC from co-culture compared to untreated PBMC. Four independent co-culture experiments for ciPTEC-U and three for ciPTEC-T1 were performed. *p < 0.001 (One-way ANOVA, Tukey’s multiple comparison test) for PBMC proliferation induced by ConA (5 µg/ml), PHA-P (5 µg/ml) or T-Activator CD3/CD28 dynabeads (1 bead per cell) compared to untreated PBMC.

The ability of ciPTEC to induce an alloimmune response was evaluated by measuring the proliferation of freshly isolated human PBMC (obtained from random blood donors) in direct co-culture experiments. The proliferative potential of the PBMC was convincingly shown after stimulation with ConA (5 µg/ml), PHA-P (5 µg/ml) and human T-Activator CD3/CD28 dynabeads (Fig. 4a and c). Notably, both ciPTEC lines failed to induce any proliferation of PBMC after 5 days of culture, irrespective of the pre-stimulation treatment of ciPTEC and ratios of ciPTEC to PBMC (Fig. 4b and c).

### Release of proinflammatory mediators by ciPTEC-U and –T1

**Figure 5:**
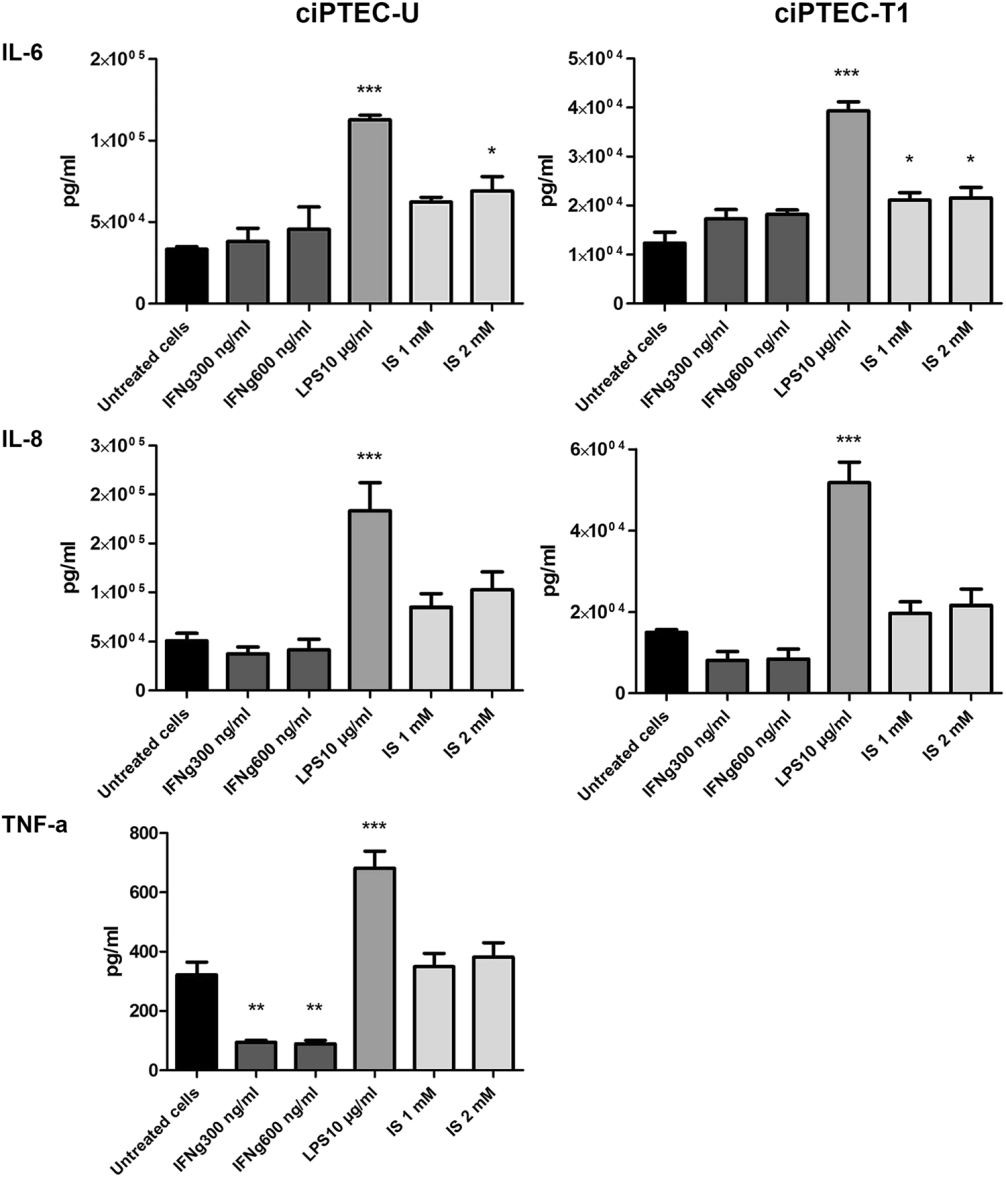
Pro-inflammatory cytokines production. Effect of 48 h exposure to IFN-γ (300 and 600 ng/ml), LPS (10 µg/ml) and indoxyl sulfate (IS) (1 and 2 mM) on proinflammatory cytokine production (IL-6, IL-8 and TNF-α) by ciPTEC-U and –T1. Concentration from three independent experiments is expressed as pg/ml (mean ± SEM). *p < 0.05, **p < 0.01, ***p < 0.001, compared to untreated cells (One-way ANOVA, Dunnett’s multiple comparison test).

To assess whether ciPTEC are able to mediate an inflammatory response, the release of IL-6, IL-8 and TNF-α was determined in cell culture supernatants (Fig. 5). In basal conditions, there was a low secretion of IL-6 and IL-8 by both cell lines, and of TNF-α by ciPTEC-U. Exposure to LPS 10 µg/ml significantly increased the secretion of all three cytokines. A moderate increase of IL-6 secretion was also observed after stimulation with indoxyl sulfate. Remarkably, TNF-α levels in supernatants of ciPTEC-U were significantly decreased after IFN-γ exposure.

### Strong ciPTEC barrier formation on microPES membranes is unaffected by allogeneic PBMC

**Figure 6:**
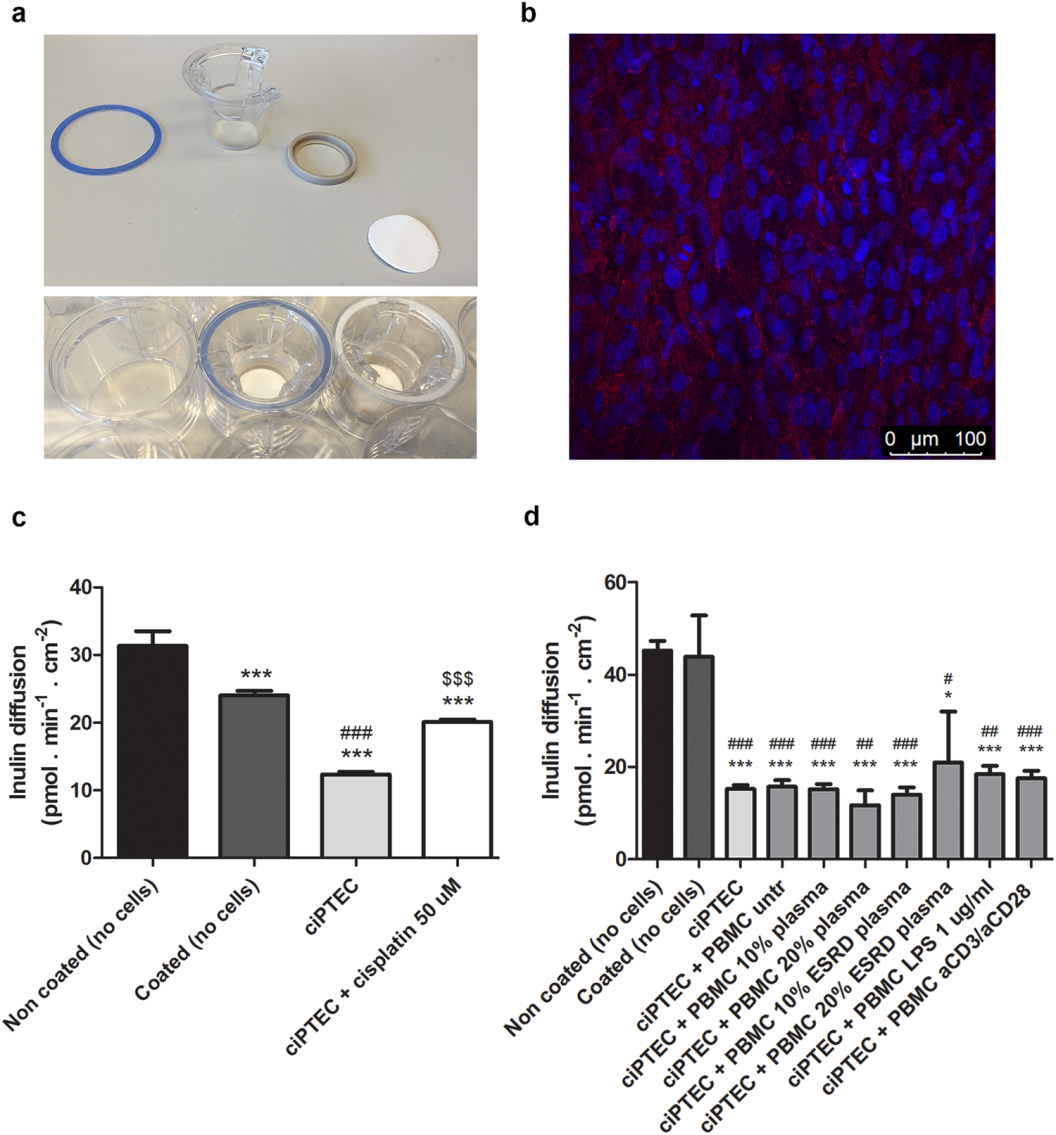
Epithelial cell monolayer formation and transepithelial barrier function following co-culture with PBMC. (**a**) Transwell® inserts and microPES hydrophilic flat membrane used for the indirect co-culture of ciPTEC-T1 and PBMC. (**b**) ZO-1 expression (red) in ciPTEC-T1 cultured on double-coated microPES membranes after 7 day maturation at 37 °C, nuclei stained with DAPI (blue) (25X magnification). (**c**) Transepithelial inulin-FITC diffusion in absence of cells, in presence of double-coating (L-DOPA 2 mg/ml and collagen IV 25 μg/ml), untreated cells and cells exposed to cisplatin 50 μM for 24 h and (**d**) Iinulin-FITC diffusion across membranes containing ciPTEC-T1 after 48 h of co-culture with PBMC in the presence of several stimulatory conditions, including plasma from ESRD patients. Diffusion is shown as pmol·min^−1^·cm^−2^ (mean ± SEM). Two independent experiments in triplicate (**c**) and three independent experiments in duplicate (**d**) were performed. *p < 0.05, ***p < 0.001, compared to non-coated membranes; ^#^p < 0.05, ^##^p < 0.01 and ^###^p < 0.001, compared to coated membranes; ^$$$^p < 0.001, compared to ciPTEC (untreated control; One-way ANOVA, Dunnett’s multiple comparison test).

Cell monolayer formation and tightness were assessed on polyethersulfone membranes, which are already in use for hemodialysis treatments and were shown to be suitable for generation of “living membranes” with functional growth of PTEC^6, 16–19^. ciPTEC-T1 were cultured on L-DOPA and collagen IV coated flat microPES membranes to grow stable cell monolayers, similarly to previously performed studies with microPES HFM^6, 12^. Figure 6a shows the Transwell®-based culture system with microsPES membranes used to generate ciPTEC monolayers, and evaluate their stability after co-culture with PBMC by measuring inulin-FITC transepithelial diffusion. The ZO-1 expression confirms that ciPTEC-T1 form tight cell monolayers on microPES membranes (Fig. 6b). Figure 6c shows inulin-FITC diffusion through untreated and cisplatin (50 μM) treated ciPTEC-T1 monolayer, indicating the specificity of barrier function. As shown in Fig. 6d, inulin-FITC diffusion through double-coated microPES membranes carrying ciPTEC-T1 was 15 ± 1 pmol·min^−1^·cm^−2^, compared to 45 ± 2 pmol·min^−1^·cm^−2^ (p < 0.001) and 44 ± 9 pmol·min^−1^·cm^−2^ (p < 0.01) for non-coated membranes and double-coated membranes without cells, respectively. Importantly, co-culturing with PBMC did not alter the barrier function, even in the presence of strong PBMC stimulators such as LPS and anti-CD3/anti-CD28, or simultaneous exposure to plasma from ESRD patients (Fig. 6d).

## Discussion

**Figure 7:**
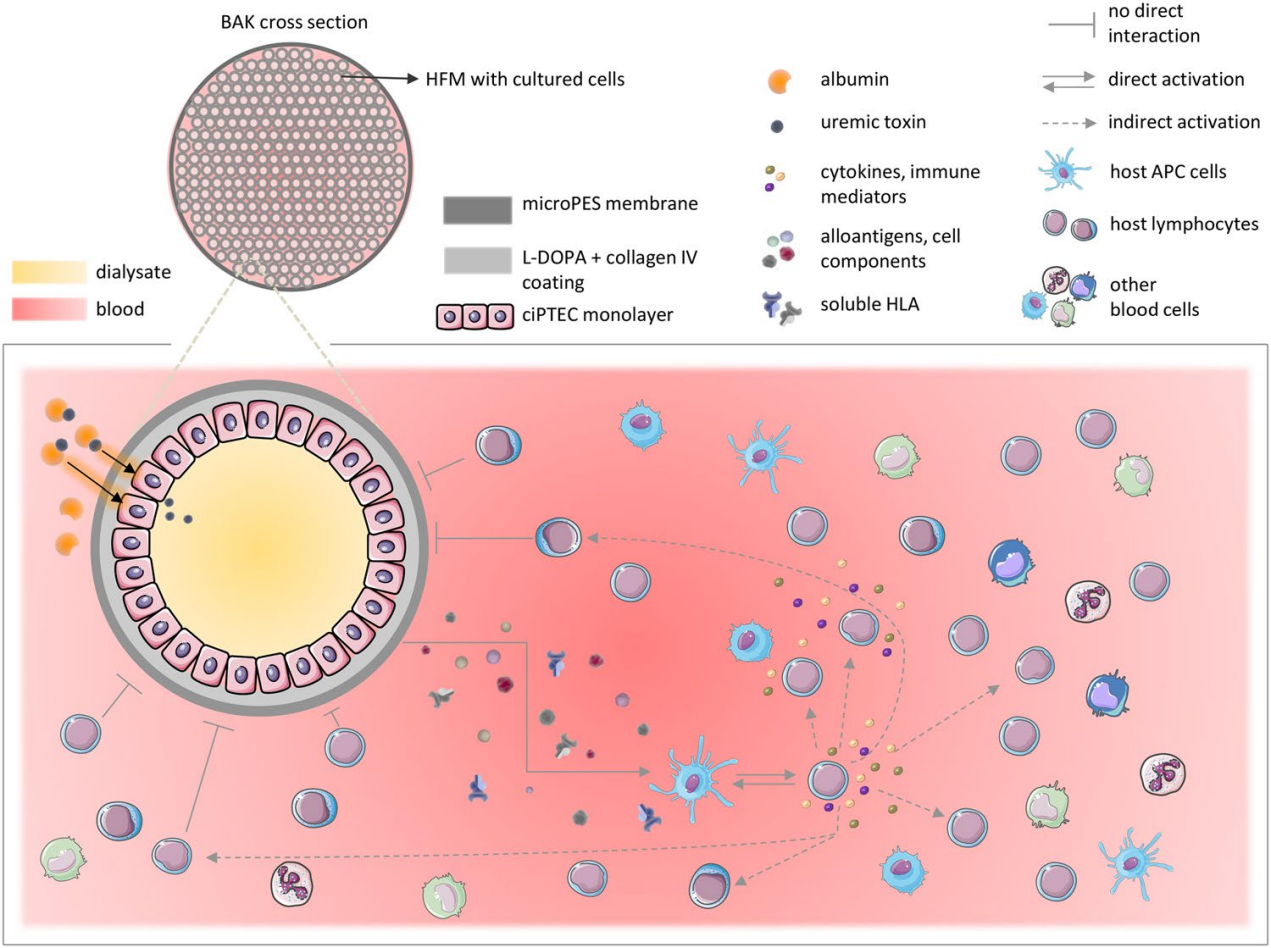
Schematic representation of BAK. The BAK device is composed of multiple HFM containing tight monolayers of ciPTEC for efficient removal of protein-bound uremic toxins. Uremic toxins bound to albumin can be taken up by ciPTEC from the blood side, and secreted into the dialysate compartment of the BAK. The presence of the polymer membrane should be sufficient to block direct interaction of host blood cells, in particular T cells, and ciPTEC via T-cell receptors and HLA molecules, thereby preventing direct activation of an immune response. However, there is a possibility of an immune response towards microvesicles, soluble allo-antigens or other cellular components that might be released by ciPTEC. In that case, it can be expected that host APC could present ciPTEC-specific antigens to helper T cells, thus leading to a semi-direct or indirect pathway of immune response activation^21^. Once activated, helper T cells can cause the activation of other cells such as cytotoxic T cells, as well as B cells and macrophages by releasing pro-inflammatory cytokines. This could potentially also lead to a non-specific inflammatory response, especially due to the excessive secretion of pro-inflammatory mediators by activated immune and inflammatory host cells.

In this study, we demonstrated the lack of immunogenic effects of ciPTEC studying several aspects of the immune system as a part of safety assessment required during the BAK device development. A prototype example of the BAK device and potential interactions with the host immune system is shown in Fig. 7. When ciPTEC lines are used in a BAK, the cells will be derived from an unrelated individual. As other nucleated somatic cells^20^, ciPTEC can be expected to express HLA class I antigens, which are highly polymorphic and able to induce alloreactive immune responses in cell, tissue, and organ transplantation. The expression of foreign HLA antigens on donor antigen presenting cells can directly activate host T cells, but the allo-antigens can also be released in microvesicles or processed into peptides and semi-directly or indirectly presented by host APC to responding T cells^21^. Here, we demonstrate that our two ciPTEC lines, urine and kidney tissue derived, indeed express HLA class I molecules on their surface, and that this expression can be increased when the cells are exposed to IFN-γ and LPS. This is in line with other studies that have described an effect of IFN-γ on HLA expression in many cell types, including renal epithelial cells^22^.

Besides the expression on cell membranes, soluble HLA molecules can be found in the circulation of healthy individuals^23^. High serum concentrations of sHLA class I molecules are related to a variety of diseases and clinical states, such as graft rejection, infection, cancer and autoimmune disease, in particular systemic lupus erythematosus and rheumatoid arthritis^24–26^. It has been described that sHLA can have immunomodulatory functions, mostly related to the induction and maintenance of self-tolerance^27, 28^. The effect of sHLA in the setting of organ transplantation is still a matter of debate^29^. In case of a bioartificial device, it can be hypothesized that sHLA is adsorbed on the polymer fibers or is taken up by antigen presenting cells, which might mediate immunomodulation in patients via interaction with T cells^30, 31^. We here showed that ciPTEC lines can release sHLA, especially after stimulation with IFN-γ as was described for other cell types^32^ and in patients with cancer^33^. The concentration of sHLA in the culture supernatant was very low compared to serum concentrations of healthy individuals or kidney transplant recipients before and after transplantation^34, 35^ for instance, or patients with hemorrhagic fever with renal syndrome^36^. Nevertheless, the production levels of sHLA observed *in vitro* can still be different from more physyiological conditions of unidirectional flow and shear stress present in the BAK.

While HLA class I antigens are expressed on the surface of almost all nucleated cells, HLA class II proteins are found mostly on the surface of professional antigen presenting cells, endothelial cells, and activated T cells^37^. We found no expression of HLA-DR antigens on ciPTEC in untreated conditions and neither after exposure to several stimuli, except for the activated PBMC-CM. This is contrary to what was observed by Demmers *et al*., who demonstrated HLA-DR expression on primary tubular epithelial cells in unstimulated conditions with an increase after IFN-γ stimulation^22^. We did not investigate further the levels of sHLA class II antigens, given the fact that HLA-DR is hardly expressed on ciPTEC and that the levels of sHLA class II proteins are usually low^38–40^.

Additionally, we investigated the expression of the co-stimulatory molecules CD40, CD80, and CD86 to evaluate the capacity of ciPTEC to act as antigen presenting cells (APC). CD40 expression has been described for many cell types, such as lymphocytes, monocytes, dendritic cells, endothelial cells, smooth muscle cells, fibroblasts, as well as epithelial cells^41^. In line with previous studies^22, 42^, we showed that CD40 is expressed in both ciPTEC lines and its expression can be enhanced in the presence of activated PBMC-CM, indicating that the particular mixture of soluble factors present in the conditioned medium can have an effect on CD40 expression. This clearly implies that CD40, in an environment that resembles the activation of the adaptive immune system, can be upregulated and be involved in further amplification of the response. CD80 and CD86, also known as B7–1 and B7-2, respectively, are the two major types of the B7 family of membrane proteins which are usually expressed on APC and other myeloid cells and provide co-stimulatory signals in the interaction between APC and T cells, via binding to CD28. A previous study suggested that CD80 and CD86 can be expressed on PTEC under certain conditions when cells are exposed to several cytokines simultaneously, such as IFN-γ, IL-1α, IL-4, IL-13, in the presence of a CD40 ligand^43^. However, we did not find any expression regardless the stimulation state, confirming the study of Demmers *et al*.^22^.

To evaluate the potential of ciPTEC to induce an allo-immune response, we performed direct co-culture experiments with ciPTEC and PBMC from healthy donors. This is the classical approach to measure the induction of cellular alloreactivity *in vitro* although the design of the BAK may not allow direct contact between ciPTEC and host immune cells (Fig. 7). We showed that ciPTEC are not able to induce the activation and proliferation of PBMC, even if they were pre-stimulated under different conditions to enhance the expression of HLA antigens and co-stimulatory molecules and, therefore, the APC phenotype. The obtained data are in line with previous studies that showed that PBMC did not proliferate after five days of direct co-culture with renal tubular epithelial cells^44^. Assays for the measurement of indirect alloreactivity are difficult and have limited reproducibility^45^. Mostly, the response induced by the direct presentation of alloantigens is stronger than that induced by indirect presentation. Therefore, the absence of a response of PBMC after co-culture with ciPTEC lines underscores the safety of the BAK device.

As described previously, PTEC are able to secrete several pro-inflammatory cytokines^46, 47^. We here confirmed that both ciPTEC lines were able to secrete IL-6 and IL-8, especially in response to LPS, which reflects an extreme inflammatory condition. TNF-α secretion was detected only in the supernatant of the urine derived ciPTEC line (ciPTEC-U) and was also strongly induced after stimulation with LPS. However, upon IFN-γ exposure the levels of TNF-α were significantly reduced, which can be due to the IFN-γ mediated downregulation of ADAM17, a metalloproteinase responsible for the cleavage of membrane-associated cytokines such as TNF-α^48^ (Supplementary Figure S1). In the context of a BAK, the production of IL-6 and TNF-α might contribute to the activation of immune cells and propagation of inflammation usually present in CKD patients^49–52^. Moreover, IL-8 could promote the chemotaxis of inflammatory cells and their eventual adhesion to the fibers containing ciPTEC. Nevertheless, when cells are cultured on HFM in 3D environment^16^, there is a polarized, apical secretion of cytokines (N. Chevtchik, M. Mihajlovic *et al*., unpublished data), suggesting that risks associated with inflammatory mediators are most likely minimal.

Besides characterizing ciPTEC and assessing their immunogenic potential, we also evaluated the effect of host immune cells on ciPTEC, resembling a possible BAK environment in which two cell populations are separated by a polymer membrane (Fig. 7). Our previous studies, as well as those from other groups, showed that PTEC can form stable and tight monolayers^6, 12, 17, 53–55^. In order to culture ciPTEC on microPES, a double coating given by L-DOPA and collagen IV is required^6, 12^. In this study, we cultured ciPTEC-T1 on flat coated microPES membranes to investigate the integrity and barrier function of cell monolayer in (patho)physiologically relevant conditions for BAK. After formation of the monolayer, ciPTEC were exposed basolaterally to PBMC, in regular as well as stimulatory conditions. Neither inflammatory stimulation of PBMC, nor a uremic-like environment provided by pooled plasma from ESRD patients, did compromise the barrier function of ciPTEC monolayer as observed by inulin-FITC transepithelial diffusion. Cisplatin was able to alter monolayer integrity, as described previously^56–58^, supporting the stability of barrier function. Such behavior of ciPTEC is very promising for the correct function of ciPTEC in BAK.

Previously, ciPTEC-U and ciPTEC-T1 lines, were characterized and compared in a study by Jansen *et al*., who showed that both types are functionally active, expressing several specific transporters while maintaining reabsorption mechanisms. The biggest difference encountered was related to a different profile of extracellular matrix (ECM) components produced by ciPTEC-U compared to ciPTEC-T1, which might explain the need for additional collagen IV coating to obtain tight monolayers^8^. In the present study, we investigated and characterized these two ciPTEC lines regarding their immunomodulatory functions and ability to act as non-professional APC. It has been suggested that epithelial cells can actually have the capacity to function as non-professional APC through the expression of HLA and co-stimulatory molecules^59^. We showed that there are no major differences between ciPTEC-U and ciPTEC-T1 with respect to the expression of HLA and co-stimulatory molecules, cytokine production and direct immunogenic effect, and neither were able to function as non-professional APC. Despite the fact that the final design of BAK is, as of yet, not completely established, the concept is to create a ciPTEC monolayer inside the HFM, for optimal function of BAK in terms of clearance. Considering that design, ciPTEC would be protected by a double-coated membrane from the blood compartment in the extracorporeal device, which would drastically, if not completely, reduce the risk of direct interaction with blood cells from the patient and immune system activation. Our data provide a first proof-of-concept encouraging further BAK development, though, a long road of research needs to be taken before such a concept can be applied in a clinical setting. Cell-engineered products, such as a BAK, need to obey guidelines as set for advanced therapy medicinal products (ATMPs) by the European Medicines Agency (EMA; Regulation EC No. 1394/2007)^60^, and the U.S. Food and Drug Administration (FDA)^61^, of which immune safety is one aspect. Other characteristics of intended cells, including the extent of replication competence of viruses, long time functionality, ability to proliferate or differentiate, the risk of oncogenicity, *in vivo* efficacy, and mode of administration or application use, have to be determined. Experiments planned in the near future will address these issues.

In conclusion, ciPTEC appear to be a safe choice for BAK application, as far as it concerns their potential to induce an alloimmune response.

## Methods

### Ethics statement

The ethics committee of the University Medical Center Utrecht on research involving human subjects approved this study, and written informed consent was obtained from each patient and each healthy volunteer. All experiments were performed in accordance with relevant guidelines and regulations.

### Cell culture

Two previously established ciPTEC lines, derived from a healthy volunteer urine sample (ciPTEC-U) and tissue from a non-transplanted donor kidney (ciPTEC-T1)^7, 8^, were cultured in Dulbecco’s Modified Eagle Medium/Nutrient Mixture F-12 (1:1 DMEM/F-12) (Gibco, Life Technologies, Paisly, UK) supplemented with 10% (v/v) fetal calf serum (FCS) (Greiner Bio-One, Alphen aan den Rijn, the Netherlands), 1% (v/v) penicillin/streptomycin (Sigma Aldrich, Zwijndrecht, the Netherlands), 5 μg/mL insulin, 5 μg/mL transferrin, 5 μg/mL selenium, 35 ng/mL hydrocortisone, 10 ng/mL epidermal growth factor and 40 pg/mL tri-iodothyronine (Sigma Aldrich, Zwijndrecht, the Netherlands). CiPTEC-U were cultured up to 50 and ciPTEC-T1 up to 45 passages. Cells were cultured at 33 °C and 5% (v/v) CO_2_ to allow proliferation, given that they were immortalized with SV40T. Prior to the experiments, cells were seeded at appropriate density, which was 48,000 cell/cm^2^ and 19,250 cell/cm^2^ for ciPTEC-U and ciPTEC-T1, respectively. Cells were grown for 1 day at 33 °C, 5% (v/v) CO_2_ to allow them to adhere, then cultured at 37 °C, 5% (v/v) CO_2_ for differentiation and maturation, changing the medium every second day. After 7 days of maturation, cells were treated with chemicals where indicated and used in experiments. The following treatments were used depending on the experimental set-up: interferon-γ (IFN- γ) 150–600 ng/ml, lipopolysaccharide (LPS) (*Escherichia coli* 0127:B8) 10 µg/ml, indoxyl sulfate 1–2 mM (all from Sigma Aldrich, Zwijndrecht, the Netherlands) for 48 h.

### HLA typing

HLA allele typing of ciPTEC cell lines was performed by the Luminex method using the LABtype SSO kit (OneLambda, Canoga Park, USA) according to the manufacturer’s instructions.

### Enzyme-Linked Immuno Sorbent Assays

The production of IL-6, TNF-α, IL-8 and soluble HLA class I (sHLA class I) molecules in various stimulatory conditions, was measured by means of Enzyme-Linked Immuno Sorbent Assays (ELISAs). Cell culture supernatants were collected after exposure to various stimulatory conditions, centrifuged for 10 min at 240 × g, 4 °C, and stored at −80 °C. DuoSet® ELISA Development Systems kits (IL-6 #DY206, TNF-α #DY210, IL-8 #DY208; R&D systems, Abingdon, UK) and HLA class I kit (Proteintech, Chicago, IL, USA) were used to quantify the cytokine and sHLA class I levels respectively, in complete cell culture medium supernatants according to manufacturer’s instructions. The optical density was determined immediately using the iMark Microplate Absorbance Reader (Bio-Rad, Japan) set to 450 nm. Each sample was measured in duplicates and quantification was done using Microplate Manager software (version 6.0, Bio-Rad Laboratories, Hercules, CA, USA) capable of generating a four parameter logistic (4-PL) curve-fit.

### Flow cytometry

The surface expression of HLA class I, HLA-DR, CD40, CD80 and CD86 molecules was evaluated using flow cytometry. First, ciPTEC were cultured until confluency (7 day maturation at 37 °C, 5% (v/v) CO_2_), followed by the stimulation with IFN-γ 150–600 ng/ml, LPS 10 µg/ml, indoxyl sulfate 1–2 mM, or conditioned medium derived from either resting or CD3/CD28-activated human peripheral blood mononuclear cells (PBMC). Next, cells were washed once with Hank’s Balanced Salt Solution (HBSS; Gibco, Life Technologies, Paisly, UK), detached with Accutase® solution (Sigma Aldrich, Zwijndrecht, the Netherlands), centrifuged at 240 × g for 5 min, washed twice with HBSS and incubated with 0.05% (v/v) viability dye (Fixable Viability Dye eFluor 780; eBioscience, San Diego, CA, USA) in protein-free Phosphate Buffered Saline (PBS) (Lonza, Verviers, Belgium) for 30 min at 4 °C, protected from light. After two washing steps with FACS buffer, an 1% (w/v) solution of Bovine Serum Albumin (BSA) (Roche Diagnostics, Mannheim, Germany) in PBS, cell pellets were fixated with ice-cold 4% (w/v) paraformaldehyde in PBS for 10 min on ice, washed, centrifuged at 240 × g for 5 min and incubated with the following antibodies diluted in FACS buffer at appropriate concentration for 30 min at room temperature (RT): anti-human HLA-DR-PE (clone LN3) (1:50), anti-human CD40-FITC (clone 5C3) (1:100), anti-human CD80(B7-1)-PE (clone 2D10.4) (1:50), anti-human CD86(B7-2)-PE (clone IT2.2) (1:20) (antibodies were purchased from eBiosciences, San Diego, CA, USA) and W6/32 for detection of human HLA class I molecules^62^ (1:200). In case of W6/32 antibody, after a washing step with FACS buffer, cell pellets were incubated with a secondary antibody (Alexa Fluor 488 goat anti-mouse IgG 1:200; Molecular probes, Eugene, OR, USA) for another 30 min in dark. Finally, after the staining procedure, cell pellets were washed and resuspended in 100–150 µl of FACS buffer and the expression of surface markers was measured using flow cytometry (BD FACSCanto II, BD Biosciences, San Jose, CA, USA). A minimum of 10,000 cells per sample were counted and data were analyzed using FlowLogic software (600.0 A; Inivai Technologies, Mentone, Victoria, Australia). Dead cells were excluded from analysis and median fluorescent intensity (MFI) was used to quantify the expression where possible.

### Isolation of peripheral blood mononuclear cells

Human PBMC were isolated from buffy coats of healthy donors (Sanquin, Amsterdam, the Netherlands) using Leucosep tubes (Greiner Bio-One, Frickenhausen, Germany). Briefly, blood was diluted 1:1 in PBS containing 2% (v/v) FCS, poured in Leucosep tubes and centrifuged (1,000 × g, 13 min). The enriched cell fraction containing PBMC was harvested and washed three times with PBS/2% (v/v) FCS. Remaining erythrocytes were lysed by adding 5 mL of cold lysis buffer (155 mM NH_4_Cl, 10 mM KHCO_3_, 0.1 mM Na_2_EDTA in 500 mL demi water, pH adjusted to 7.4 and filter sterilized) for 5 min on ice. Afterwards, PBMC were resuspended in Roswell Park Memorial Institute (RPMI) 1640 medium (Lonza, Verviers, Belgium) supplemented with 10% (v/v) FCS, 1% (v/v) penicillin/streptomycin and 1 mM sodium pyruvate (Sigma Aldrich, Zwijndrecht, the Netherlands). Conditioned medium from PBMC was collected after 24 h of either unstimulated cells, or cells stimulated with anti-CD3 (clone CLB-T3/2) and anti-CD28 (clone CLB-CD28) antibodies (both 1:10,000, Sanquin), and used diluted 1:1 with RPMI 1640 medium.

### Direct co-culture of ciPTEC and PBMC

Following 7 days of maturation at 37 °C, 5% (v/v) CO_2_, ciPTEC were stimulated with IFN-γ, LPS or exposed to conditioned medium from CD3/CD28-activated PBMC for 48 h. Prior to the co-culture, freshly isolated PBMC were labelled with carboxyfluorescein diacetate succinimidyl ester (CFDA-SE or CFSE) (Vybrant® CFDA-SE Cell Tracer Kit, Life Technologies, Eugene, OR, USA), 5 µM final concentration. In brief, the staining was done in PBS for 10 min, at RT, in dark. The reaction was quenched with ice-cold FCS, after which the cells were washed three times with PBS/2% (v/v) FCS and resuspended in RPMI 1640. In the meantime, ciPTEC were rinsed with HBSS, detached using Accutase® solution and resuspended at appropriate concentration in RPMI 1640. CiPTEC-PBMC co-cultures were performed in 96 well U-bottom cell culture plates (Greiner Bio-One, Frickenhausen, Germany) at different ratios (1:5, 1:15 and 1:30 – ciPTEC:PBMC) for at least 5 days. As positive controls CFSE-labelled PBMC were stimulated with concanavalin A (ConA) (Sigma Aldrich, Zwijndrecht, the Netherlands) 5 µg/ml, phytohemagglutinin-P (PHA-P) (Sigma Aldrich, Zwijndrecht, the Netherlands) 5 µg/ml, or with human T-Activator CD3/CD28 dynabeads (Gibco, Thermo Fisher Scientific, Vilnius, Lithuania), at a ratio of one bead per cell. Proliferation of CFSE-labelled PBMC was assessed using flow cytometry by measuring dilution of CFSE and therefore cell division^63^. Data were analyzed using FlowLogic software and presented as percentage of cells in the divided population^64^.

### Cell culture on microPES and co-culture of ciPTEC-T1 and PBMC

MicroPES (polyethersulfone) type 2 F iv hydrophilic flat membranes (thickness 110 ± 10 μm) were purchased from 3 M Deutschland GmbH (Wuppertal, Germany). For cell culture, round-shaped pieces of the microPES membranes (diameter 12 mm, surface growth area 1.12 cm^2^) were cut from the flat sheets, mounted on empty Transwell®(Corning Costar, NY, USA) membrane support systems using custom-made sealing rings^53^, sterilized with 0.2% (v/v) solution of peracetic acid (Sigma Aldrich, Zwijndrecht, the Netherlands) in 4% (v/v) ethanol for 30 min, and then extensively rinsed with HBSS. Afterwards, the double coating was applied on the membranes to support cell attachment and growth, based on previously published studies^12, 17^. First, L-DOPA (L-3,4-dihydroxyphenylalanine, Sigma Aldrich, Zwijndrecht, the Netherlands) was dissolved in 10 mM Tris buffer (pH 8.5) at 37 °C for 45 min with occasional mixing, at 2 mg/ml final concentration, as described previously^6, 12, 65^, filter sterilized and applied on the membranes for 4 h at 37 °C. The second coating was provided by 25 μg/ml solution of collagen IV (Sigma Aldrich, Zwijndrecht, the Netherlands), for 1 h at 37 °C. Following the coating procedure, microPES membranes were washed in HBSS and used further for cell seeding.

CiPTEC-T1 were seeded on double-coated membranes, in the apical compartment of the Transwell® system, at 90,000 cells/cm^2^. After initial proliferation at 33 °C for 1 day, and 7 days maturation at 37 °C, the indirect co-culture of 48 h was performed with freshly isolated PBMC which were seeded in the basolateral compartment (2 million cells/ml), and exposed to anti-CD3 and anti-CD28 antibodies (both 1:10,000), LPS (1 μg/ml), or human plasma pooled from healthy donors and from CKD patients (10% (v/v) or 20% (v/v), diluted with culture medium).

### Cell monolayer integrity

Following 48 h indirect co-culture, PBMC were removed and ciPTEC-T1 cell monolayer barrier function on double-coated microPES membranes was assessed by quantifying diffusion of inulin-fluorescein isothiocyanate (FITC) (Sigma Aldrich, Zwijndrecht, the Netherlands) (0.1 mg/ml in Krebs-Henseleit (KH) buffer (Sigma Aldrich, Zwijndrecht, the Netherlands) supplemented with 10 mM HEPES (Acros Organics, New Jersey, USA)) from basolateral to apical compartment for 1 h at 37 °C. Coated and non-coated membranes without cells were used as controls. Apical and basolateral exposure of ciPTEC-T1 to cisplatin (Sigma Aldrich, Zwijndrecht, the Netherlands; 50 μM) for 24 h was used as a positive control for inulin-FITC leakage. Fluorescence was measured at excitation wavelength of 492 nm and emission wavelength of 518 nm, by means of fluorescent platereader (Fluoroskan Ascent FL, Labsystems). Measured fluorescence values were used to calculate inulin-FITC concentration in apical compartment of all samples. Finally, inulin-FITC diffusion (*J*) was calculated as described previously^12^, using the following formula:

$$J=((((C/Mw)\ast V)\ast {10}^{9})/t)/A=pmol\cdot mi{n}^{-1}\cdot c{m}^{-2}$$

where *C* is the apical concentration of inulin-FITC in mg/ml, *Mw* the average inulin-FITC molecular weight (4,500 mg/mmol), *V* the volume in the apical compartment (0.5 ml), *t* the time (60 min) and *A* the surface area of microPES membranes (1.12 cm^2^).

### Immunocytochemistry

The expression of zonula occludens-1 (ZO-1) on ciPTEC-T1 monolayers cultured on microPES membranes was assessed using a slightly modified immunocytochemistry procedure described by Jansen *et al*.^12^. In brief, cells were washed with HBSS two times, fixed with 2% (w/v) paraformaldehyde in PBS containing 4% (w/v) sucrose (Sigma Aldrich, Zwijndrecht, the Netherlands) for 5 min, then washed three times with 0.1% (v/v) Tween (Sigma Aldrich, Zwijndrecht, the Netherlands) solution in PBS and permeabilized with 0.3% (v/v) Triton (Merck, Darmstadt, Germany) solution. After another three washing steps with 0.1% (v/v) Tween-PBS, cells were exposed to Dako Target Retrieval solution (DAKO, Carpinteria, CA, USA) for 1 h, according to manufacturer’s instructions. Next, cells were incubated with DAKO Protein Block serum-free solution (DAKO, Carpinteria, CA, USA) for 1 h, followed by the application of rabbit anti-human ZO-1 antibody (Invitrogen, Carlsbad, CA, USA) (1:400 in DAKO Antibody Diluent (DAKO, Carpinteria, CA, USA)) for 1 h at RT. After extensive washing with 0.1% (v/v) Tween-PBS, secondary antibody (Alexa Fluor 568 goat anti-rabbit IgG 1:200 in DAKO Antibody Diluent; Life Technologies, Eugene, OR, USA) was applied for 1 h at RT, followed by incubation with phalloidin-FITC (1:500 in PBS) (Sigma Aldrich, Zwijndrecht, the Netherlands) for 30 min at RT to stain actin filaments. Finally, membranes were mounted on glass slides using ProLong Gold antifade reagent containing DAPI (Life Technologies, Eugene, OR, USA) and cells were imaged using confocal microscope (Leica TCS SP8 X, Leica Microsystems CMS GmbH, Wetzlar, Germany) and analyzed using Leica Application Suite X software (Leica Microsystems CMS GmbH).

### Data analysis

All data are presented as mean ± standard error of the mean (SEM). Statistical analysis was performed using one-way ANOVA followed by Dunnett’s multiple comparison test or, where appropriate, Tukey’s multiple comparison test, and a p-value < 0.05 was considered significant. Software used for statistical analysis was GraphPad Prism (version 5.03; GraphPad software, La Jolla, CA, USA). All experiments were repeated independently at least three times, unless stated differently.

## Electronic supplementary material

Supplementary Information
