# Supplementary material for: Allostimulatory capacity of conditionally immortalized proximal tubule cell lines for bioartificial kidney application
Source: Sci Rep. 2017 Aug 2;7:7103. doi: 10.1038/s41598-017-07582-1 (PMC5540916; doi:10.1038/s41598-017-07582-1)
Supplement: Supplementary file 1 — Supplementary Information [file 41598_2017_7582_MOESM1_ESM.pdf]

Allostimulatory capacity of conditionally immortalized proximal tubule cell lines for bioartificial kidney application

Supplementary material

Milos Mihajlovic[1], Lambertus P. van den Heuvel[2], Joost G. Hoenderop[3], Jitske Jansen[1], Martijn J. Wilmer[4], Annemarie J.F. Westheim[1], Wil A. Allebes[5], Dimitrios Stamatialis[6], Luuk B. Hilbrands[7†] and Rosalinde Masereeuw[1†*]

[1]Division of Pharmacology, Utrecht Institute for Pharmaceutical Sciences, Utrecht University, Utrecht, The Netherlands

[2]Department of Pediatric Nephrology, Radboud university medical center, Nijmegen, The Netherlands

[3]Department of Physiology, Radboud Institute for Molecular Life Sciences, Radboud university medical center, Nijmegen, The Netherlands

[4]Department of Pharmacology and Toxicology, Radboud Institute for Molecular Life Sciences, Radboud university medical center, Nijmegen, The Netherlands

[5]Department of Laboratory Medicine, Laboratory for Medical Immunology (LMI), Radboud university medical center, Nijmegen, The Netherlands

[6]Department of Biomaterials Science and Technology, MIRA Institute for Biomedical Technology and Technical Medicine, University of Twente, Enschede, The Netherlands

[7]Department of Nephrology, Radboud university medical center, Nijmegen, The Netherlands

† Both authors contributed equally

* Corresponding author:
Rosalinde Masereeuw, Ph.D.
Utrecht University
Div. Pharmacology
Department of Pharmaceutical Sciences
Universiteitsweg 99
3584 CG Utrecht
The Netherlands
r.masereeuw@uu.nl
Phone: +31-30-253-3529
Fax: +31-30-253-7900

Supplementary Table S1: PCR-SSO typing of HLA isotypes of two ciPTEC cell lines. The HLA-A, -B and –C alleles were examined in MHC class-I locus and HLA-DRB, -DQA and –DQB in MHC class-II locus.

	HLA-I			HLA-II		
	HLA-A	HLA-B	HLA-C	HLA-DRB	HLA-DQA	HLA-DQB
ciPTEC-U	A*01	B*07	C*07:01	DRB1*03	DQA1*01	DQB1*02
	A*24	B*08	C*07:02	DRB1*15	DQA1*05:01	DQB1*06
ciPTEC-T1	A*02	B*07	C*03:04	DRB1*11	DQA1*01	DQB1*03:01
	A*03	B*40:01	C*07:02	DRB1*13	DQA1*05	DQB1*06

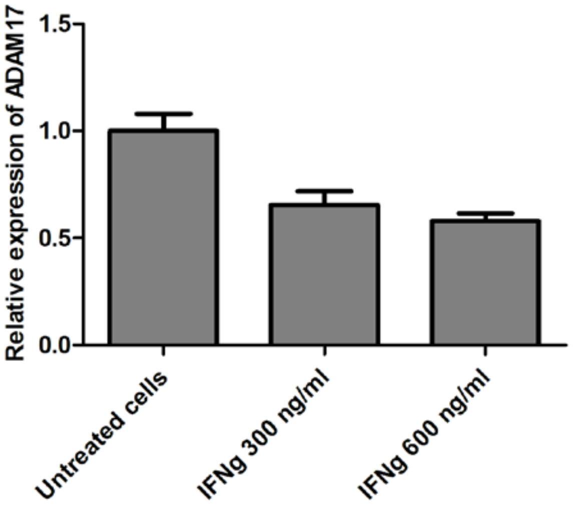

Supplementary Figure S1: Relative gene expression of ADAM17 in ciPTEC-U after 48 h exposure to IFN-γ (300 and 600 ng/ml). GAPDH used as a house-keeping reference gene. Experiment was performed once in duplicates. Specific sense and anti-sense primers for ADAM17 (forward:

TCCAGCAGCATTCGGTAAGAA; reverse: AGAGTCAGGCTCACCAACCA) and GAPDH (forward: ACAGTCAGCCGCATCTTCTT; reverse: ACGACCAAATCCGTTGACTC) were synthesized by Biolegio (Nijmegen, The Netherlands). Gene expression was performed by total RNA isolation using RNeasy Mini kit (Qiagen, Venlo, The Netherlands) according to the manufacturer's instructions, followed by cDNA synthesis using the Omniscript RT-kit (Qiagen, Venlo, the Netherlands) and Real-Time PCR using the iQ SYBR® Green Supermix (Bio-Rad Laboratories, Hercules, CA, USA). The data were analyzed using Bio-Rad CFX Manager™ Software version 3.1 (Bio-Rad Laboratories, Hercules, CA, USA).
